# Combination of an Autophagy Inducer and an Autophagy Inhibitor: A Smarter Strategy Emerging in Cancer Therapy

**DOI:** 10.3389/fphar.2020.00408

**Published:** 2020-04-08

**Authors:** Ting Liu, Jing Zhang, Kangdi Li, Lingnan Deng, Hongxiang Wang

**Affiliations:** ^1^The Central Hospital of Wuhan, Tongji Medical College, Huazhong University of Science and Technology, Wuhan, China; ^2^College of Life Sciences, Wuhan University, Wuhan, China; ^3^Department of Digestion, The Second Affiliated Hospital of Jiangxi University TCM, Nanchang, China

**Keywords:** autophagy inducer, cytoprotective autophagy, autophagy inhibitor, drug resistance, cancer therapy

## Abstract

Autophagy is considered a cytoprotective function in cancer therapy under certain conditions and is a drug resistance mechanism that represents a clinical obstacle to successful cancer treatment and leads to poor prognosis in cancer patients. Because certain clinical drugs and agents in development have cytoprotective autophagy effects, targeting autophagic pathways has emerged as a potential smarter strategy for cancer therapy. Multiple preclinical and clinical studies have demonstrated that autophagy inhibition augments the efficacy of anticancer agents in various cancers. Autophagy inhibitors, such as chloroquine and hydroxychloroquine, have already been clinically approved, promoting drug combination treatment by targeting autophagic pathways as a means of discovering and developing more novel and more effective cancer therapeutic approaches. We summarize current studies that focus on the antitumor efficiency of agents that induce cytoprotective autophagy combined with autophagy inhibitors. Furthermore, we discuss the challenge and development of targeting cytoprotective autophagy as a cancer therapeutic approach in clinical application. Thus, we need to facilitate the exploitation of appropriate autophagy inhibitors and coadministration delivery system to cooperate with anticancer drugs. This review aims to note optimal combination strategies by modulating autophagy for therapeutic advantage to overcome drug resistance and enhance the effect of antitumor therapies on cancer patients.

## Highlights

1. A number of agents in development and even clinical drugs for cancer treatment have cytoprotective autophagy effects that contribute to drug resistance.
2. Combination treatment with an autophagy inducer and inhibitor is a good opportunity for discovery and development of more novel and effective therapeutic approaches for cancer treatment.

## Introduction

Macroautophagy (hereafter termed autophagy) is a physiological and dynamic process dependent on the formation of double-membrane vesicles to maintain metabolic homeostasis by capturing intracellular constituents, including redundant or unnecessary proteins, injured or aged organelles, and later degrading them in lysosomes. Basal autophagy is widely accepted as a mechanism of cell survival under conditions of nutrient deprivation because the lysosome-released breakdown products are recycled into metabolic and biosynthetic pathways (White et al., 2015). Autophagy is also a cytoprotective mechanism against various environmental stresses, such as oxidant stress, endoplasmic reticulum stress, and viral or bacterial infection, by eliminating damaged and toxic cellular components and products. However, autophagy plays a dual role in both tumor-suppressing and -promoting activity in cancer initiation, development, progression, and treatment by preventing the toxic accumulation of oncogenic signaling substances from carcinogenic factors, such as genomic injury to suppress cancer initiation. By contrast, cancer cells tend to utilize autophagy-mediated recyclable biomolecules to meet the increased metabolic energy demand of survival and proliferation and take advantage of the engulfment capability to overcome micro-environmental stress, which facilitates tumorigenesis and aggressiveness (Galati et al., 2019). It has been shown that cancer cells are more autophagy-dependent than normal tissues. Thus, targeting autophagy directly is a therapeutic strategy for cancer therapy (White, 2015).

Autophagy in cancer treatment is also context-dependent and complicated. There are generally two effects of autophagy in response to anticancer drugs or ionizing radiation treatment in cancer cells (Thorburn et al., 2014). One effect is the cytotoxic function known as autophagic cell death, also named type II programmed cell death (Fulda and Kögel, 2015; Denton and Kumar, 2019). It is a nonapoptotic form of programmed cell death caused by overactivated autophagy (Booth et al., 2019; Zhu et al., 2020). Anticancer treatment induces robust autophagy of cancer cells to self-digestion until death (Simonet et al., 2020; Ganesher et al., 2020). Many natural compounds and synthetic agents exhibit their anticancer effects through triggering autophagic cell death (Law et al., 2017; Xu et al., 2019; Kiruthiga et al., 2020; Pellerito et al., 2020). Moreover, the activation of autophagy-related signaling may implicate the suppression of certain other cancer therapeutic target to help against cancer, such as tumor invasion and migration and tumor angiogenesis (Wang et al., 2019; Jiang et al., 2019; Song et al., 2019).

The other effect is the cytoprotective function, which is a drug resistance mechanism resulting in a clinical obstacle to successful cancer treatment and leads to a poor prognosis of the patients. The cancer cells initiate autophagy to escape from the damage of drugs or radiation. Efforts to inhibit treatment-induced autophagy has therefore attracted great interest to improve cancer therapy efficiency (Nagelkerke et al., 2015). By combining the antineoplastic agents, the application of autophagy inhibitors is considered beneficial to increase the susceptibility of cancer cells to therapeutic agents that induce autophagy (Dalby et al., 2010; Kleger et al., 2014). Thus, it is critical to determine if anticancer drugs and radiation treatment actually promote cytoprotective autophagy in patient tumors (G, 2014).

Multiple agents in development in preclinical and clinical trials and even many clinical drugs have been found to trigger cytoprotective autophagy, including mTOR inhibitors, kinase inhibitors, natural products, and antiangiogenic agents (Haiyang Yu et al., 2019; Mei-Chuan Chen et al., 2019). Meanwhile, various signaling pathways and molecules have been identified in regulating drug-induced autophagy that impact the outcome of anticancer therapy, such as the PI3K-Akt-mTOR pathway, one of the main regulators of autophagy. The anticancer drug-triggered DNA damage may play a crucial role in the initiation of autophagy signaling cascades to elevate DNA repair levels and promote cellular survival (Zhang et al., 2015). Research has demonstrated autophagy as a universal cytoprotective response after DNA damage induced by chemotherapeutic drugs, including cisplatin, BO-1051, and doxorubicin (DOX) in hepatocellular carcinoma cell lines (Chen et al., 2011). High mobility group box 1 (HMGB1) is associated with the hallmarks of cancer. Autophagy-associated HMGB1 has been revealed to protect various cancer cells, such as osteosarcoma, lung adenocarcinoma, neuroblastoma and ovarian cancer, from many chemotherapeutics, including DOX, cisplatin and etoposide (Wang et al., 2015). HMGB1-mediated autophagy through the mitogen-activated protein kinase (MEK)/extracellular signal-regulated kinase (ERK) signaling pathway promotes docetaxel resistance in human lung adenocarcinoma (Pan et al., 2014). HMGB1 release is also a key regulator of autophagy and promotes tumor resistance to chemotherapy in leukemia (Liu et al., 2011). Additionally, in gastric cancer cells, after vincristine, a microtubule-targeting drug treatment, HMGB1 released into the extracellular space to protect cancer cells from apoptosis by upregulating the transcription of Mcl-1 (Zhan et al., 2012). VEGF-C/NRP-2 axis is another signaling pathway involved in autophagy activation through inhibition of mTOR complex 1 activity, results in aiding cancer cell survival under therapeutic treatment (Stanton et al., 2013). Protective autophagy regulated by PP2Ac and ERK are at least parts of the mechanism that contribute to cisplatin resistance in certain ovarian cancer cells (Yin et al., 2013; Wang and GS, 2014). The autophagic response regulated by JNK-Bcl-2 pathway plays a role in limiting the anticancer activity and toxicity of CA-4 in clinical application for various cancers. Thus, a JNK inhibitor or a Bcl-2 inhibitor (ABT-737) could promote CA-4-elicited apoptosis due to inhibition of autophagy (Li et al., 2014). Effective targeting of these pathways may intervene in therapy to render cancer cells resistant to cell inhibition, such as cell death, cell proliferation and tumor angiogenesis, as well as leading to the development of novel cancer therapies.

## Autophagy Inhibitors

Not surprisingly, increasing research has demonstrated that drug resistance in cancer therapy can be abrogated by the inhibition of autophagy *via* genomic interference against autophagic genes (siRNA targeting Atg3, Atg5, Atg7, and Beclin 1) or pharmacological inhibitors of key components within the autophagy pathway in cancer resistance (Kumar et al., 2015) (**Table 1**). Additionally, there is also a growing interest in exploring more potent and specific pharmacological autophagy inhibitors (Golden et al., 2015; Li et al., 2016).

**Table 1:** Autophagy inhibitors in cancer cells.

Autophagy inhibitor	Target point	Inhibition stage	References
**3-MA**	PI3K inhibition	Early	(Horwacik et al., 2015; Zhang et al., 2015)
**LY294002**	PI3K/mTOR inhibition	Early	(Feng et al., 2018; Shen et al., 2017)
**Baf A1**	Vacuolar-type H(+)-ATPase inhibition	Late	(Miyazawa, 2011)
**CQ**	Lysosomal inhibition	Late	(Kimura et al., 2012; Fukuda et al., 2015; Liu et al., 2016)
**HCQ**	Lysosomal inhibition	Late	(Vogl et al., 2014; Rangwala et al., 2014)
**ABT-737**	Bcl-2 inhibition	Early	(Huang and Sinicrope, 2010; Yang et al., 2016)
**Obatoclax**	Lysosomal inhibition	Late	(Koehler et al., 2015; Jiménez-Guerrero et al., 2018)
**Clarithromycin**	Block autophagy flux	Late	(Altman and Platanias, 2012; Sugita et al., 2015)
**Resveratrol**	Autophagy regulator -S6K1	Early	(Lin et al., 2012; Alayev et al., 2015; Rai et al., 2016)
**Quinacrine**	Lysosomal inhibition	Late	(Lobo et al., 2013; Golden et al., 2015)
**4-Acetylantroquinonol B**	Block autophagy flux	Late	(Liu et al., 2017)
**EGCG**	Block autophagy flux	Late	(Meng et al., 2019)

3-MA, 3-methyladenine; Baf A1, bafilomycin A1; PI3K, phosphatidylinositol 3 kinase; mTOR, mammalian target of rapamycin; CQ, chloroquine; HCQ, hydroxychloroquine; ATPase, adenosine triphosphatase; EGCG, Epigallocatechin gallate.

3-Methyladenine (3-MA), LY294002, and Bafilomycin A1 (Baf A1) are common autophagy inhibitors that function in early autophagy by PI3K inhibition and in late autophagy by blocking vacuolar-type H(+)-ATPase, respectively (Feng et al., 2018; Yang et al., 2013). Chloroquine (CQ), a 4-alkylamino-substituted quinoline family member, is a clinically available antimalarial agent which is also an autophagy inhibitor function by blocking the fusion of autophagosomes and lysosomes. Because CQ has been approved by the U.S. Food and Drug administration (FDA) for use as an antimalarial agent, it is commonly used in clinical trials as an inhibitor of autophagy (Kimura et al., 2012; Kuroda et al., 2013), as well as another more toxic safety agent for autophagy inhibition, hydroxychloroquine (HCQ) (Wolpin et al., 2014).

Except the classical autophagy inhibitors mentioned above, more agents with function of autophagy inhibition in certain cases have been proved. This kind of autophagy inhibitors may have multiple biological activities and exhibit promising inhibitory effect of therapy-induced cytoprotective autophagy that facilitate overcoming acquired resistance to antitumor therapy. The Bcl-2 family of proteins are not only regulators of apoptosis signaling, but also involved in autophagy processes (Duffy et al., 2015). The Bcl-2 inhibitor ABT-737 has been identified as an autophagy inhibitor (Yang et al., 2016). Moreover, obatoclax which exhibits pan-Bcl-2 inhibition effect has been demonstrated to cause a striking inhibition of autophagy at late-stage in colorectal cancer and bladder cancer cells (Huang and Sinicrope, 2010; Koehler et al., 2015; Jiménez-Guerrero et al., 2018). Clarithromycin is a macrolide antibiotic frequently utilized in the treatment of upper and lower respiratory tract infections and Helicobacter pylori that has demonstrated inhibitory effects on autophagy (Altman and Platanias, 2012; Giulia Petroni et al., 2020). Resveratrol is a natural polyphenolic compound derived from plants which has proapoptotic effects on various cancer cells. Interestingly, resveratrol showed synergistic anticancer efficacy by blocking temozolomide or doxorubicin induced cytoprotective autophagy flux (Lin et al., 2012; Rai et al., 2016). Quinacrine also known as mepacrine which is a synthetic antimalarial drug belonging to the quinoline-based drugs class and have been demonstrated to inhibit autophagy at late stage (Golden et al., 2015). 4-Acetylantroquinonol B is a novel compound derived from antroquinonol by the addition of an acetyl group. It had been first demonstrated by a group that it could enhance the drug susceptibility of the epithelial cancerous cells to cisplatin by inhibition of autophagic flux (Liu et al., 2017). Epigallocatechin gallate (EGCG) is a bioactive catechin derived from green tea which has been employed to overcome drug resistance by inhibiting therapy-induced autophagic flux (Meng et al., 2019).

## Antitumor Agents With Cytoprotective Autophagy

Accumulating evidence has shown that targeting autophagy in combination with antitumor agents has been effective at enhancing cell death and improving the efficacy of cancer therapies in various cancer types. The antitumor agents are currently under investigation with a cytoprotective autophagy effect that is mainly classified into several types according to distinct characteristic (**Table 2**).

**Table 2:** Subset of the preclinical research targeting drug-induced autophagy in various cancers.

Drug Classification	Drug name	Targeting cancer cells	Autophagy inhibitor	References
**Natural compounds**	Polyphyllin I Ursolic acid Paclitaxel Tetrandrine Pterostilbene Topotecan Cucurbitacin Sulforaphane Honokiol Combretastatin A-4	Hepatoma Prostate cancer Cervical cancer Renal cancer Bladder cancer Various cancers Lung cancer Breast cancer Lung cancer Glioblastoma Neuroblastoma Lung cancer Various cancers	CQ 3-MA 2-deoxyD-glucose 3-MA or BafA1 Obatoclax CQ 3-MA or BafA1 3-MA or BafA1 CQ CQ 3-MA CQ ABT-737, 3-MA or BafA1	(Shi et al., 2015) (Shin et al., 2012; Junco et al., 2015) (Peng et al., 2014) (Zhang et al., 2013) (Jiménez-Guerrero et al., 2018) (Mei et al., 2015) (Hsieh et al., 2013) (Wei-Chih Chen et al., 2014) (Wang Y et al., 2011) (Yuan et al., 2014) (Horwacik et al., 2015) (Lv et al., 2015) (Li et al., 2014)
**Tyrosine kinase inhibitor**	Imatinib Sorafenib Sunitinib Linifanib Gefitinib Erlotinib Cediranib	Leukemia Glioblastoma Hepatoma Glioblastoma Various cancers Hepatoma Breast cancer Lung cancer Lung cancer Glioblastoma	CQ or Clarithromycin BafA1 3-MA CQ CQ CQ, HCQ or 3-MA HCQ, BafA1 or 3-MA Clarithromycin, EGCG CQ Quinacrine	(Altman and Platanias, 2012; Zeng et al., 2015) (Shingu et al., 2009) (Yuan et al., 2014) (Liu et al., 2016) (Abdel-Aziz et al., 2014) (Pan et al., 2014) (Dragowska et al., 2013; Liu et al., 2017) (Sugita et al., 2015; Meng et al., 2019) (Zou et al., 2013) (Lobo et al., 2013)
**Conventional cytotoxic drugs**	Cisplatin Oxaliplatin Temozolomide 5-Fluorouracil Cytarabine Doxorubicin Pirarubicin	Lung cancer Ovarian cancer Glioblastoma Gastric cancer Bladder cancer Endometrial cancer Epithelial cancer Colorectal cancer Glioblastoma Cholangiocarcinoma Colon cancer Acute myeloid Hepatoma, osteosarcoma leukemia Osteosarcoma Breast cancer Bladder cancer	3-MA or CQ 3-MA or CQ 3-MA CQ 3-MA or CQ CQ 4-Acetylantroquinonol B 3-MA Resveratrol or CQ Quinacrine Capsaicin 3-MA or CQ Baf A1 or CQ EGCG 3-MA Resveratrol HCQ or 3-MA HCQ or 3-MA	(Wu et al., 2015; Liu et al., 2015) (Zhang et al., 2012; Wang and GS, 2014; Bao et al., 2015) (Zhang et al., 2015) (Zhang et al., 2015) (Ojha et al., 2016) (Fukuda et al., 2015) (Liu et al., 2017) (Liu et al., 2015) (Lin et al., 2012; Yan et al., 2016) (Buccarelli et al., 2018) (Hong et al., 2015) (Sasaki et al., 2010; Li et al., 2010) (Bosnjak et al., 2014) (Chen et al., 2014; Wang and Ding Chen, 2018) (Zhao et al., 2014) (Rai et al., 2016) (Pan et al., 2015) (Li et al., 2015)
**Proteasome inhibitor**	Carfilzomib	Myeloma	CQ, HCQ	(Jarauta et al., 2016; Baranowska et al., 2016)
	Bortezomib	Myeloma Myeloma	Macrolide antibiotics BafA1, HCQ	(Moriya et al., 2013) (Di Lernia et al., 2020; Miyazawa, 2011)
	Ixazomib	Glioblastoma Colorectal cancer	3-MA ABT-737	(Zhang et al., 2014) (Yang et al., 2016)

CQ, chloroquine; HCQ, hydroxychloroquine; 3-MA, 3-methyladenine; Baf A1, bafilomycin A1; EGCG, Epigallocatechin gallate.

### Natural Compounds

Multiple plant-derived natural compounds have obvious anticancer potential. However, autophagy-associated chemoresistance limits the development of novel natural drugs in clinical cancer treatment (Zhang et al., 2014; Guo et al., 2015). Polyphyllin I (PPI) is a bioactive phytochemical isolated from the rhizoma of *Paris polyphyllin*. Preclinical studies revealed PPI has anticancer efficacy with autophagy induction in various cancer models. Further combined PPI with CQ to block PPI-induced autophagy in HCC cells resulted in augmenting the cytotoxicity and antiproliferation effects of PPI *via* the caspase-dependent apoptosis pathway (Shi et al., 2015). By the Beclin-1 and Akt/mTOR pathway, ursolic acid (UA), a pentacyclic triterpenoid derived from natural plants, showed an autophagic response as a survival mechanism in PTEN-deficient PC3 prostate cancer cells. Blockade of autophagy by 3-MA enhanced UA-induced apoptosis (Shin et al., 2012). Additionally, UA and resveratrol have been shown to synergize with CQ to enhance melanoma cell death (Junco et al., 2015). By interfering with the normal breakdown of microtubules during cell division, paclitaxel is a medication used to treat several cancer types, including breast cancer, lung cancer and ovarian cancer. Acquired resistance mediated by autophagy of paclitaxel functions as a major obstacle to successful anticancer effects. 2-Deoxy-D-glucose or 3-MA could enhance the preferential toxicity on paclitaxel resistant HeLa cervical cancer cells *via* decreasing autophagy (Peng et al., 2014). Moreover, the blockade of autophagy with 3-MA and Baf A1 strengthen sensitivity of folliculin-deficient renal cancer cells to paclitaxel (Zhang et al., 2013). Obatoclax could also promote paclitaxel induced apoptosis in synergistic manner by blockade of the autophagic flux in bladder cancer (Jiménez-Guerrero et al., 2018). Tetrandrine is a natural product study in our laboratory, and we found that tetrandrine combined with CQ has synergistic antitumor activity (Mei et al., 2015). It was also reported that pterostilbene in combination with 3-MA or BafA1 may enhance the efficiency of chemotherapeutic approaches in both chemo-sensitive and chemo-resistant lung cancer cells and in triple-negative breast cancer cells (Hsieh et al., 2013; Wei-Chih Chen et al., 2014). The anticancer effect of another natural substance product, chaetocin, is enhanced by Baf A1(Jung et al., 2016). Moreover, CQ potentiated the cytotoxicity of topotecan in lung cancer cells by interfering with autophagy (Wang Y et al., 2011), and the antitumor efficiency of cucurbitacin I is promoted with synergetic treatment of CQ in glioblastoma (Yuan et al., 2014). Additionally, cell death of BE (2)-C human neuroblastoma cells following sulforaphane treatment could be promoted by 3-MA *via* inhibition of autophagy (Horwacik et al., 2015). Honokiol is isolated from the bark, seed cones, and leaves of trees belonging to the genus Magnolia and is a kind of lignan. Honokiol-induced cell death increased with CQ by inhibiting autophagy that finally exhibits augmented antitumor effects in human nonsmall cell lung cancer cells (Lv et al., 2015). Combretastatin A-4 (CA-4) is a drug isolated from combretum caffrum which has been applied in clinical trials for solid tumors therapy in past over ten years. However, the CA-4-elicited autophagic response in various cancer cells restricts its clinical application. Autophagy inhibition by autophagy inhibitors (3-MA and Baf A1), the JNK inhibitor or the Bcl-2 inhibitor ABT-737 could promote CA-4-induced apoptosis (Li et al., 2014).

### Synthetic Compounds

#### Conventional Cytotoxic Drugs

Cytotoxic drugs are used in the treatment of tumors to trigger the death of tumor cells by preventing DNA replication and cell division. Cisplatin-based chemotherapy frequently results in acquired resistance, which is a major challenge in the clinical control of various cancers. The underlying mechanism is demonstrated in relation to the autophagic response. Combined treatment of cisplatin with 3-methyladenosine or CQ promotes the chemotherapeutic sensitivity of various cancers, including lung cancer, ovarian cancer, glioma cancer, gastric cancer, bladder cancer, and endometrial cancer cells (Zhang et al., 2012; Wang and GS, 2014; Bao et al., 2015; Wu et al., 2015; Zhang et al., 2015; Zhang et al., 2015; Fukuda et al., 2015; Ojha et al., 2016). Coadministration of CQ and cisplatin to abolish the suppression of mTORC1 activity-mediated autophagy significantly re-sensitized cisplatin-resistant EC109/CDDP cells (Yu et al., 2014). 4-Acetylantroquinonol B can also act as an autophagy inhibitor by blocking autophagic flux and improving the sensitivity of highly aggressive epithelial cancer to cisplatin *via* the PI3K/Akt/mTOR/p70S6K signaling pathway (Liu et al., 2017). Another platinum-based antineoplastic agent, oxaliplatin, shows the drug resistance *via* the MEK/ERK signaling pathway and HMGB1-mediated autophagy in colorectal cancer cells, and the sensitivity can be restored by 3-MA (Liu et al., 2015). Temozolomide (TMZ) is an alkylating agent used for the clinical treatment of glioblastoma multiforme and melanoma. Studies have revealed that cytoprotective autophagy induced by TMZ contributes to therapy resistance in malignant glioma which can be suppressed by resveratrol, chrysin, or CQ and its analog quinacrine, resulting in a decrease in autophagy and an increase in apoptosis (Buccarelli et al., 2018; Lin et al., 2012; Yan et al., 2016). 5-Fluorouracil (5-FU) is a pyrimidine analog for cancer treatment that works through irreversible inhibition of thymidylate synthase. Capsaicin is a major pungent ingredient found in hot red chili peppers of the genus capsicum, emerges as a chemotherapeutic augmenter for 5-FU's anticancer effects in cholangiocarcinoma (Hong et al., 2015). In addition, CQ and 3-MA potentiate the cytotoxic effect of 5-fluorouracil on colon cancer cells (Sasaki et al., 2010; Li et al., 2010). Cytarabine is a chemotherapy agent used mainly in killing acute myeloid leukemia (AML) and non-Hodgkin lymphoma cancer cells by interfering with DNA synthesis. Baf A1 and CQ can markedly increase apoptotic death in cytarabine-treated human leukemic cells (Bosnjak et al., 2014).

Anthracycline drugs derived from Streptomyces bacterium Streptomyces peucetius var. caesius, which are used in cancer chemotherapy to treat several cancers, including breast, ovarian, uterine, bladder, lung cancers, and leukemias, and lymphomas. Doxorubicin (DOX) is an anthracycline antibiotic derived by chemical semisynthesis from bacterial species and works by intercalating DNA for the treatment of various cancers. EGCG, one of the highest catechins from green tea, promisingly showed the capability to augment the antitumor efficacy of DOX in HCC and osteosarcoma treatment involving autophagy inhibition (Chen et al., 2014; Wang and Ding Chen, 2018). As a classic chemotherapeutic agent for osteosarcoma, autophagy-mediated resistance of DOX can be reversed by 3-MA (Zhao et al., 2014). Resveratrol can enhance the chemotherapeutic potential of DOX by inducing apoptosis mediated through down regulation of autophagy in breast cancer cell lines (Rai et al., 2016). Additionally, HCQ or 3-MA can partially reverse the drug resistance of myeloma RPMI8226/DOX cells by inhibition of autophagy (Pan et al., 2015). Pirarubicin is widely used in clinical chemotherapy for bladder cancer. However, emerging evidence has shown that the efficacy of pirarubicin is limited by the cytoprotective role of autophagy in bladder cancer cells, and inhibition of autophagy by 3-MA or HCQ increased cell apoptosis, suggesting an efficiency over traditional pirarubicin chemotherapy in bladder cancer patients (Li et al., 2015).

#### Tyrosine Kinase Inhibitors

Tyrosine kinases play a pivotal role in oncogenesis, but emerging studies have report compromised cytotoxicity of tyrosine kinase inhibitors used as monotherapy in cancer (Carew et al., 2008). Imatinib (INN) is a frontline tyrosine-kinase inhibitor notably used in the targeted therapy of Philadelphia chromosome-positive (Ph+) chronic myelogenous leukemia (CML) by targeting BCR-Abl-expressing leukemic cells. Autophagy induction has been identified as the imatinib resistance mechanism during therapeutic process. CQ could markedly promote CML cell apoptosis induced by Hedgehog pathway suppression of imatinib-sensitive or -resistant BCR-ABL+ cells (Zeng et al., 2015). Additionally, clarithromycin, which blocks autophagy, could also restore the sensitivity of CML cells to imatinib (Altman and Platanias, 2012). In human malignant glioma cells, the imatinib-elicited cytotoxicity has been enhanced by induction of apoptosis with Baf A1 which trigger inhibition of autophagy at a late stage (Shingu et al., 2009). Sorafenib, which is a multikinase inhibitor that inhibits serine/threonine kinases and receptor tyrosine kinases (RTKs), was shown to have survival benefits in advanced HCC. Inhibition of cytoprotective autophagy by 3-MA treatment enhances sorafenib-mediated cell death *via* necrosis and significantly augments the combination antitumor effect with sorafenib and the HDAC inhibitor vorinostat in hepatocellular carcinoma cells (Honma and Harada, 2013; Yuan et al., 2014). Additionally, sorafenib has shown antitumor activity in glioblastoma multiforme (U373 and LN229 cells). Further study demonstrated that combination treatment with sorafenib and CQ exhibited inhibition of cell proliferation and migration and induction of cell apoptosis by blockade of autophagy *in vitro* and *in vivo* (Liu et al., 2016). Sunitinib is a multitargeted receptor tyrosine kinase inhibitor that was approved for the treatment of renal cell carcinoma and imatinib-resistant gastrointestinal stromal tumor. CQ has been shown to synergize with sunitinib by switching off autophagy then enhanced the cytotoxicity of sunitinib *via* inducing apoptosis (Abdel-Aziz et al., 2014). Linifanib (ABT-869) is a structurally novel and potent multikinase inhibitor of RTK, vascular endothelial growth factor (VEGF), and platelet-derived growth factor. Autophagy was found to impair the sensitivity of HCC cells to linifanib-targeted therapy by the suppression of Akt/mTOR and Mek/Erk signaling pathways and CQ, HCQ, or 3-MA greatly augments the anti-HCC effect of linifanib (Pan et al., 2014). Gefitinib is a small-molecule inhibitor of epidermal growth factor receptor (EGFR) tyrosine kinase used for certain breast, lung, and other cancers with mutated and overactive EGFR. HCQ or Baf A1 inhibit gefitinib-induced autophagy at late-stage significantly increased cell death in gefitinib-sensitive and -insensitive breast cancer cells (Dragowska et al., 2013). 3-MA or Baf A1 also improved the sensitivity of gefitinib to MDA-MB-231 and MDA-MB-468 triple-negative breast cancer cells (TNBCs) (Liu et al., 2017). In addition, the autophagy flux inhibitor clarithromycin (CAM), a macrolide antibiotic can enhances the cytotoxic effect of gefitinib in nonsmall cell lung cancer cells (NSCLC), as well as EGCG can overcomes NSCLC resistance to gefitinib by inhibiting autophagy and augmenting cell death through targeting ERK pathway (Sugita et al., 2015; Meng et al., 2019). The antagonistic activity on cell proliferation has been found when coadministration of gefitinib and cisplatin to EGFR-TKI-sensitive human lung cancer PC9 cells. After combination with CQ, resulted in a synergistic effect *via* inhibiting autophagy, further suggesting a potential strategy to reverse the antagonistic effects between EGFR-TKIs and chemotherapeutic drugs (Liu et al., 2015). Another EGFR-TK inhibitor erlotinib has been demonstrated to trigger autophagy in wild-type EGFR NSCLC. Drug resistance caused by this autophagy can be overcame with CQ which represent a beneficial strategy to enlarge the application scope of erlotinib efficacy in cancer therapy (Zou et al., 2013). Cediranib is a potent inhibitor of VEGF receptor tyrosine kinases. Combined with the late-stage autophagy inhibitor quinacrine, the antiangiogenic efficacy of cediranib in intracranial glioma is synergistically enhanced (Lobo et al., 2013).

#### Proteasome Inhibitors

Proteasome inhibitors has been widely used as clinical anticancer drugs for the bone marrow cancer multiple myeloma (MM) and exhibited remarkable efficacy in solid tumor malignancies treatment, including the first-in-class proteasome inhibitor bortezomib and second-in-class proteasome inhibitors carfilzomib and oprozomib (Saavedra-García et al., 2020). However, increasing studies indicate that cancer cells show resistance to the proteasome inhibitors and autophagy contributes to the mechanisms associated with carfilzomib and bortezomib resistance (Zang et al., 2012; Zheng et al., 2017). CQ and HCQ can enhance carfilzomib induced cell apoptosis by inhibition of autophagy toward MM (Jarauta et al., 2016; Baranowska et al., 2016). The cytotoxicity of bortezomib on MM also can be augmented by Baf1, HCQ, or macrolide antibiotics *via* inhibiting prosurvival autophagy in cotreatment manner (Di Lernia et al., 2020; Miyazawa, 2011; Moriya et al., 2013). Moreover, 3-MA promoted sensitivity of glioblastoma cells to bortezomib by inhibition of bortezomib induced cytoprotective autophagy (Zhang et al., 2014). MLN9708, the active form is ixazomib which is an orally administered proteasome inhibitor. The cytotoxic effect of ixazomib on colorectal cancer cells has been proved enhanced with ABT-737 by autophagy inhibition *via* inhibiting Mcl-1 expression (Yang et al., 2016).

#### Other Specific Signaling Inhibitors

The PI3K/Akt/mTOR pathway plays a pivotal role in oncogenesis; consequently, it is an attractive pharmacologic target. Because mTOR inhibition is involved in the induction of autophagy that limits the therapeutic effects of PI3K/Akt/mTOR signaling inhibitors, autophagy inhibition can overcome antitumor therapeutic resistance to PI3K/Akt/mTOR signaling inhibitors. Rapamycin is an allosteric mTORC1 inhibitor that has been identified as a broad-spectrum autophagy inducer. Combination therapy of rapamycin with resveratrol by autophagy blockade showed enhanced cell apoptosis effects in breast cancer cells (Alayev et al., 2015). Everolimus, a mTOR inhibitor, has been approved for second-line therapy. Combination everolimus/CQ could strongly and synergistically induce renal cancer cell death (Grimaldi et al., 2015). In addition, WYE-354, a novel mTORC1/2 dual inhibitor, of which the potential anticolon cancer cell activity can be augmented by Baf A1 and 3-MA treatment (Wang et al., 2016). Baf A1, 3-MA and CQ can also enhance a novel mTOR kinase inhibitor, KU-0063794, which induces cytotoxicity against anti-HepG2 hepatocellular carcinoma cells (Yongxi et al., 2015). The hydroxymethylglutaryl-coenzyme A (HMG-CoA) reductase-inhibiting drug simvastatin induces the activation of AMP-activated protein kinase (AMPK) and mTOR in glioma cell death. Inhibition of autophagy with Baf A1 and 3-MA, as well as AMPK inhibition with compound C, markedly increased simvastatin-induced apoptotic death (Misirkic et al., 2012). By inhibiting HMG-CoA reductase, statins can also mediate cytoprotective autophagy in human leukemic cells, and Baf A1 enhanced the apoptotic death induction (Vilimanovich et al., 2015). In aggressive prostate cancers, the efficacy of the AKT inhibitor AZD5363 is limited, and blocking autophagy using 3-MA, CQ, and Baf A1 enhanced cell death (Lamoureux et al., 2013). Pharmacological Notch1 signaling blockade by the γ-secretase inhibitor MRK003 is used to treat glioblastoma neurospheres, and combination treatment with CQ can abrogate chemoresistance caused by induction of protective autophagy (Natsumeda et al., 2016). The addition of CQ could also enhance the cytotoxic effects of flavopiridol, a cyclin-dependent kinase (CDK) inhibitor, in chronic lymphocytic leukemia (CLL) (Mahoney et al., 2012). Store-operated Ca^2+^ entry (SOCE) inhibitor SKF-96365 exhibits potent antineoplastic activity, but its antitumor capacity is often limited by cytoprotective autophagy that delays apoptosis. HCQ could significantly augment the anticancer effect of SFK-96365 in colorectal cancer (Jing et al., 2016). Dichloroacetate (DCA), an inhibitor of pyruvate dehydrogenase kinase (PDK), was demonstrated to be a promising nontoxic antineoplastic agent. DCA-induced protective autophagy can be inhibited by 3-MA and then restore DCA-induced apoptosis in LoVo colonic carcinoma cells (Gong et al., 2013) (**Table 3**).

**Table 3:** Autophagy inhibition of drugs targeting specific signaling in cancer treatment.

Drug name	Targeting signaling	Autophagy Inhibitor	Targeting Cancer cells	References
**Rapamycin**	mTORC1	Resveratrol	Breast cancer	(Alayev et al., 2015)
**Everolimus**	PI3K/AKT/mTOR	CQ	Renal cancer	(Grimaldi et al., 2015)
**Simvastatin**	AMPK	BafA1, 3-MA or AMPK Inhibitor	Glioblastoma	(Misirkic et al., 2012)
**SKF-96365**	Calcium/CaMKIIγ/AKT	HCQ	Colorectal cancer	(Jing et al., 2016)
**AZD5363**	AKT	BafA1, 3-MA or CQ	Prostate cancer	(Lamoureux et al., 2013)
**AZ7328**	AKT	CQ	Bladder cancer	(Dickstein et al., 2012)
**MRK003**	Notch1/γ-secretase	CQ	Glioblastoma	(Natsumeda et al., 2016)
**Flavopiridol**	CDK	CQ	Leukemia	(Mahoney et al., 2012)
**SKF-96365**	SOCE	HCQ	Colorectal cancer	(Jing et al., 2016)
**Dichloroacetate**	PDK	3-MA	Colorectal cancer	(Gong et al., 2013)

CQ, chloroquine; HCQ, hydroxychloroquine; 3-MA, 3-methyladenine; Baf A1, bafilomycin A1; PI3K, phosphatidylinositol 3 kinase; mTORC1, mammalian target of complex 1; Akt, protein kinase B; mTOR, mammalian target of rapamycin; AMPK, adenosine monophosphate activated protein kinase; CDK, cycling-dependent kinase; SOCE, store-operated Ca^2+^ entry; PDK, pyruvate dehydrogenase kinase.

## Clinical Trials

Based on preclinical research, including ***in vitro*** and ***in vivo*** models, the researchers conducted several clinical trials, combining drugs that triggered protective autophagy with an autophagy inhibitor (Poklepovic and Gewirtz, 2014) (**Table 4**).

**Table 4:** Combination treatment by targeting drug-induced autophagy in clinical trials for cancer therapy.

Phase	Drug name	Targeting cancer cells	Autophagy inhibitor	References
I	Temozolomide	Melanoma and advanced solid tumors	HCQ	(Rangwala et al., 2014)
I/II	Temozolomide	Glioblastoma	HCQ	(Rosenfeld et al., 2014)
I	Doxorubicin	Advanced solid tumors	Pantoprazole	(Brana et al., 2014)
I	Doxorubicin	Lymphoma	HCQ	(Barnard et al., 2014)
I	Bortezomib	Myeloma	HCQ	(Vogl et al., 2014)
I	Temsirolimus	Melanoma	HCQ	(Rangwala et al., 2014)
I	Vorinostat	Advanced solid tumors	HCQ	(Mahalingam et al., 2014)

HCQ, hydroxychloroquine.

In patients with advanced solid tumors and melanoma, a phase I trial of HCQ with dose-intense temozolomide was conducted (Rangwala et al., 2014). Another phase I/II trial concerning the antitumor activity of HCQ with temozolomide and radiation for glioblastoma patients was performed, accompanied by pharmacodynamic and pharmacokinetic analyses for HCQ dose-dependent autophagy inhibition (Amaravadi et al., 2011; Rosenfeld et al., 2014). A phase I trial in patients with advanced solid tumors conducted based on preclinical models showed that a proton pump inhibitor, pantoprazole exerted enhanced antitumor activity of DOX by improving drug distribution and inhibiting autophagy (Brana et al., 2014). In addition, a phase I clinical trial was conducted along with pharmacodynamics evaluation of combination treatment with HCQ and DOX for spontaneously occurring lymphoma in pet dogs (Barnard et al., 2014). Using combined autophagy inhibitor HCQ and proteasome inhibitor bortezomib, a phase I trial in patients with relapsed/refractory myeloma was conducted (Vogl et al., 2014). Combination treatment with mTOR and autophagy inhibitor, a phase I trial of temsirolimus and HCQ in patients with advanced solid tumors and melanoma was conducted (Rangwala et al., 2014). Moreover, coadministration with HCQ and HDAC inhibitor vorinostat, a phase I trial in patients with advanced solid tumors was conducted and researchers further analyzed the data from the effect of safety, tolerability, pharmacokinetics, and pharmacodynamics (Mahalingam et al., 2014).

## The Challenge and Development of Targeting Cytoprotective Autophagy as a Cancer Therapeutic Approach in Clinical Application

Since autophagy was considered as a double-edged sword that might cooperate, aggravate, or antagonize apoptosis, current understanding of the role of autophagy in the response to some certain cancer therapy remains controversial, such as sorafenib- induced autophagy in HCC. The effect of sorafenib-triggered autophagy serves as a prosurvival response or promotes the lethality of sorafenib against HCC cells is indistinct (Liu et al., 2017). Therefore, targeting autophagy induced by antitumor agents by combination of these agents with autophagy inhibitors in clinical cancer therapy application to improve outcomes for patients remains a challenge. More preclinical research should be conducted to confirm that autophagy provides an adaptive mechanism of resistance to antitumor drugs in cancer cells and inhibition of autophagy could further enhance the cytotoxic effects of these drugs in a synergistic manner rather than attenuating the autophagy-dependent antitumor effect.

It is clear that using autophagy inhibitor to target antitumor agents-induced protective autophagy to augment cytotoxic effects *via* switching-off the autophagic mechanism, coadministration is the key procedure. Antitumor agents with cytoprotective autophagy and autophagy inhibitors are different in physicochemical properties, such as molecular weight and size. If given separately, may result in differences in bio-distribution and cancer cell accumulation between the two drugs, which means failure combination treatment. Thus, a drug delivery system needed to facilitate the combination strategy, especially for some agents cannot enter cells efficiently by itself. By taking advantage of nanoparticles, researchers successfully coloaded miR-375, an inhibitor of autophagy and sorafenib into calcium carbonate nanoparticles with lipid coating (miR-375/Sf-LCC NPs) followed by significant autophagy inhibition and enhanced antitumor effect of sorafenib in HCC (Pengxuan Zhao et al., 2018). Another group prepared R8-dGR peptide modified paclitaxel (PTX) and HCQ incorporating liposomes (PTX/HCQ - R8- dGR- Lip) for increasing drug delivery and confirmed that combined chemotherapeutic PTX with HCQ exhibited augmented efficiency on inhibiting malignant melanoma (Sheng Yin et al., 2018). In addition, researchers developed “a strategy based on Cu(I)-catalyzed click chemistry-triggered aggregation of azide/alkyne-modified micelles for the codelivery of the DOX and the autophagy inhibitor wortmannin.” This Dox/wortmannin coloaded size-adjustable micelles exerted a significant antitumor effect in a synergistic manner in melanoma and breast cancer by inhibition of autophagy (Jingdong Rao et al., 2019).

Besides, autophagy also found as a survival pathway to induce therapy resistance in sonodynamic therapy (SDT) for several cancers. Therefore, targeting autophagy regulation is also a strategy to enhance SDT efficiency. For breast cancer, a biomimetic nanoplatform exhibited capability in restoring cells' sensitivity to SDT *via* autophagy inhibition. This design “based on hollow mesoporous titanium dioxide nanoparticles by HCQ loading and cancer cell membrane coating” (Qianhua Feng et al., 2019). For the chemotherapy of glioma, blood brain barrier and autophagy-induced chemo-resistance are two limitation factors. One group designed a smart “all-in-one” nanosensitizer platform to improve therapeutic efficiency by coloading the sonoactive chlorin e6 (Ce6) and HCQ into angiopep-2 peptide-modified liposomes (Fei Qu et al., 2019).

Clinically used antimalarial drugs CQ and its derivative HCQ, are well-known autophagy inhibitors function by preventing the acidification of the lysosomal compartment. Although CQ and HCQ showed an equipotent effect at autophagy inhibition *in vitro* studies, the toxicity of them showed different *in vivo*. High peak concentrations of CQ may result in infant deaths in case reports that associated with single-tablet ingestions indicated the significant toxicity of CQ. However, people survived suicide attempts taking HCQ, demonstrating HCQ is safely dose-escalated in cancer patients. Moreover, clinical trials showed that autophagy is unable to be completely inhibited by CQ *in vivo*. These warrant us that searching more potent autophagy inhibitors is critical for promoting an opportunity to apply the combination therapeutic strategy in clinical studies (Lalita Guntuku et al., 2019), especially agents that specifically inhibit autophagy-related (ATG) proteins (Pei-Feng Liu1 et al., 2018).

**Figure 1:**
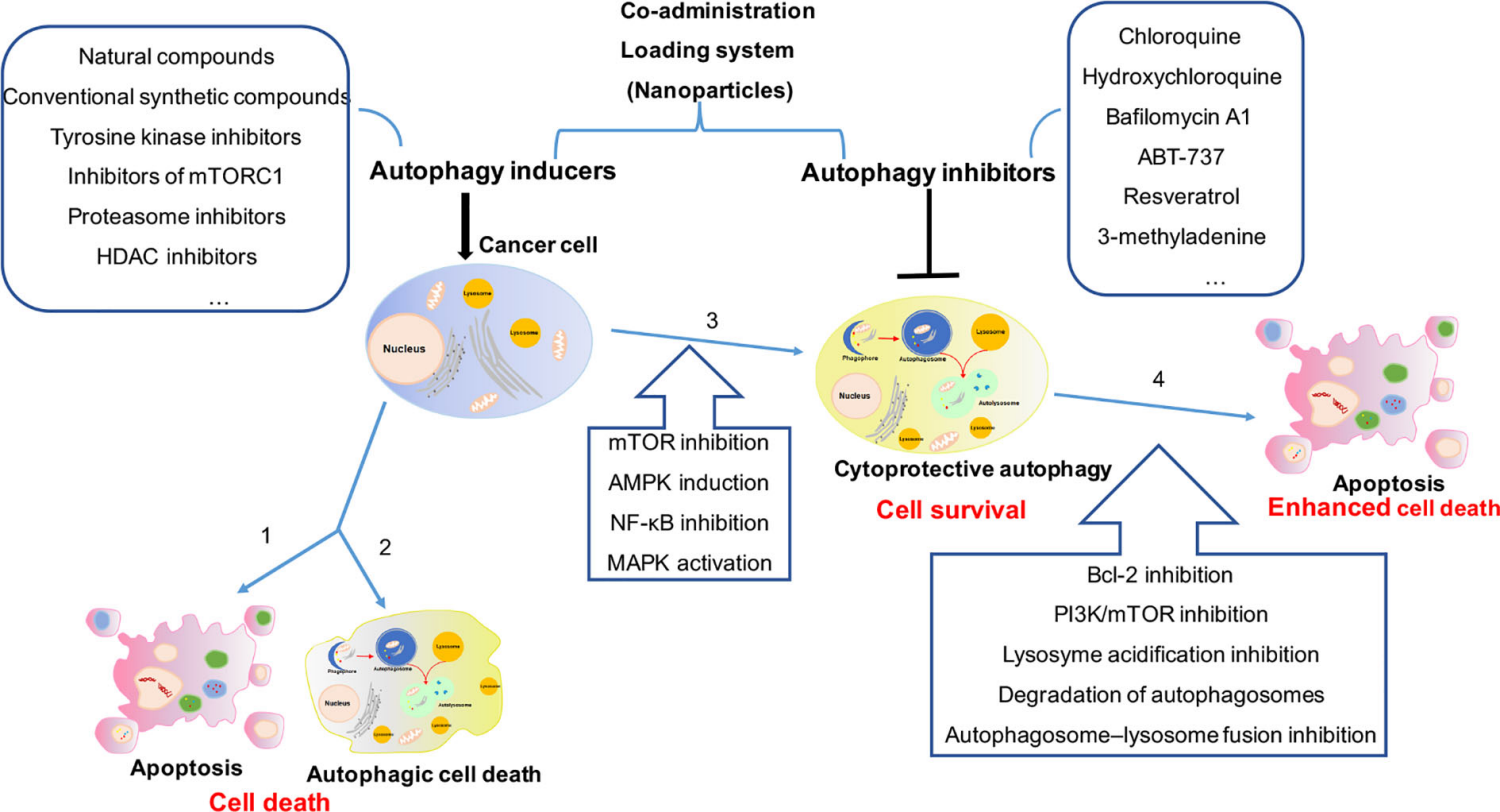
The comprehensive mechanism of autophagy induction and inhibition in cancer therapy. (1) Cancer cells treated with autophagy inducers alone will trigger cell death as 1 apoptosis and/or 2 autophagic cell death, or will induce 3 cytoprotective autophagy result in drug resistance. (2) Combination treatment with autophagy inducers and inhibitors in cancer cells will 4 enhance cytotoxicity by cytoprotective autophagy inhibition. (3) More efforts should be made in identifying the role of autophagy induced by cancer therapy (2 or 3), developing beneficial coadministration system for drug delivery and discovering novel and efficient autophagy inhibitors.

Targeting the autophagic process by coupling autophagy inhibitors with current cytotoxic chemotherapy or other available anticancer therapies are really considered promising therapeutic strategy for cancer. For further clinical application, more efforts should be made in identifying the role of autophagy induced by cancer therapy, developing beneficial coadministration system for drug delivery and discovering novel and efficient autophagy inhibitors (**Figure 1**).

## Discussion

Autophagy may act as one of the contributing factors in cellular mechanism of survival during cancer development and therapeutically triggered stress with basic biological importance. Thus, basic and clinical research will be imperatively needed to identify if autophagy is a cancer treatment-related resistance mechanism (Tan et al., 2015; Sannigrahi et al., 2015; Hu et al., 2016).

Studies in preclinical models established autophagy as a therapeutic target, and inhibition of autophagy enhanced chemo-sensitivity and promoted tumor cell death.

Meanwhile, multiple early-phase clinical trials evaluated the effect of autophagy inhibition by using HCQ combined with therapeutic agents. These studies indicated that strategies targeting autophagy in cancer are potentially new opportunities for drug development, thus more potent autophagy inhibitors are needed to identify (Yang et al., 2011). CD133 on cancer stem cells is considered a potential therapeutic target. There has been a report that CD133 mAb can sensitize HCC cells to DOX and cisplatin attributed to inhibiting autophagy and facilitating necrotic cell death. Therefore, targeting CD133 with the autophagy inhibitor CD133 mAb is a potential therapeutic approach for hepatocellular carcinomas (Chen et al., 2013). It is easier for solid tumor cells to trigger cell autophagy against nutrient deprivation and oxygen stress conditions caused by antiangiogenic therapy. Thus, we could exploit combination treatment with autophagy inhibitors and antiangiogenic agents, such as bevacizumab (Guo et al., 2013).

For most antitumor agents, combination treatment with either autophagy initiation inhibitors or autophagy maturation inhibitors causes synergistic effects (Adiseshaiah et al., 2013). However, in some circumstances, combination treatment with different autophagy inhibitors at different stages of autophagy may lead to opposite effects. Using 3-MA or small interfering RNA against Atg5 (siRNA-ATG5) to suppress imatinib-induced autophagy at an early stage could decrease the cytotoxicity of imatinib. By contrast, inhibiting autophagy at a late stage by Baf A1 augmented imatinib-induced apoptosis mediated through mitochondrial disruption, indicating that the cytotoxic efficiency of imatinib for malignant glioma may only be enhanced by the appropriate autophagy inhibitor function at a late stage of autophagy (Shingu et al., 2009). Similarly, inhibition of the early stages of autophagy by 3-MA attenuated the cytotoxic effect of arsenic trioxide (ATO) in glioblastoma multiforme cells. By contrast, interfering autophagy flux at a late stage by CQ enhanced the ATO induced cell death (Li et al., 2015). Both pharmacological and molecular targeting of elements of the autophagic process need to occur under certain circumstances to apply to therapeutic approaches.

Autophagy also acts as an obstacle in radiotherapy (Ye et al., 2016). Based on this notion, a strategy was designed to block autophagy in tumor cells to augment radio-sensitization by the autophagy inhibitor 3-MA (Chen et al., 2011). Except for cancer cells, autophagy also plays a central role in the function of immune cells. Anticancer agents with metabolic modulating by autophagy induction can result in antitumor immune disorders (Townsend et al., 2012). It has been reported that inhibiting autophagy during interleukin 2 (IL-2) immunotherapy can promote long-term tumor regression by tumor inhibition and enhance immune cell proliferation and infiltration (Liang et al., 2012). Thus, combination radiation or chemotherapy with an autophagy inhibitor can both selectively suppress tumor cells and restore the capability of the immune system by promoting the integrity of beneficial antitumor immune cells (Gewirtz, 2014).

This review was aimed to identify a potent strategy to enhance the sensitivity of cancer cells to therapeutic agents. All sections and tables are not meant to be completely presented because many compounds and agents have effect of autophagy regulation by autophagy induction or inhibition in cancer therapy, and new ones are being discovered routinely.

## Author Contributions

TL designed and wrote the manuscript. JZ revised the article KL and LD collected the material. HW supervised and directed all work.

## Funding

This study was funded by the Hubei Science and Technology Program (2018CFB116), Project of Wuhan Municipal Health Commission (WZ18Q02) and National Natural Science Foundation of China (81903101).

## Conflict of Interest

The authors declare that the research was conducted in the absence of any commercial or financial relationships that could be construed as a potential conflict of interest.

## References

[B1] Abdel-Aziz A. K. Shouman S. El-Demerdash E. Elgendy M. Abdel-Naim A. B. (2014). Chloroquine synergizes sunitinib cytotoxicity via modulating autophagic, apoptotic and angiogenic machineries. Chem. Biol. Interact. 217, 28–40. 10.1016/j.cbi.2014.04.007 24751611

[B2] Adiseshaiah P. P. Clogston J. D. McLeland C. B. Rodriguez J. Potter T. M. Neun B. W. (2013). Synergistic Combination Therapy with Nanoliposomal C6- Ceramide and Vinblastine is Associated with Autophagy Dysfunction in Hepatocarcinoma and Colorectal Cancer Models. Cancer Lett. 337 (2), 254–265. 10.1016/j.canlet.2013.04.034 23664889 PMC3722309

[B3] Alayev A. Berger S. M. Kramer M. Y. Schwartz N. S. Holz a. (2015). The Combination of Rapamycin and Resveratrol Blocks Autophagy and Induces Apoptosis in Breast Cancer Cells. J. Cell Biochem. 116 (3), 450–457. 10.1002/jcb.24997 25336146 PMC4491987

[B4] Altman J. K. Platanias L. C. (2012). A new purpose for an old drug: inhibiting autophagy with clarithromycin. Leuk Lymphoma 53 (7), 1255–1256. 10.3109/10428194.2012.661857 22288786

[B5] Amaravadi R. K. Lippincott-Schwartz J. Yin X. M. Weiss W. A. Takebe N. Timmer W. (2011). Principles and current strategies for targeting autophagy for cancer treatment. Clin. Cancer Res. 17 (4), 654–666. 10.1158/1078-0432.CCR-10-2634 21325294 PMC3075808

[B6] Bao L. Jaramillo M. C. Zhang Z. Zheng Y. Yao M. Zhang D. D. (2015). Induction of autophagy contributes to cisplatin resistance in human ovarian cancer cells. Mol. Med. Rep. 11 (1), 91–98. 10.3892/mmr.2014.2671 25322694 PMC4237096

[B7] Baranowska K. Misund K. Starheim K. K. Holien T. Johansson I. Darvekar S. (2016). Hydroxychloroquine potentiates carfilzomib toxicity towards myeloma cells. Oncotarget 7 (43), 70845–70856. 10.18632/oncotarget.12226 27683126 PMC5342593

[B8] Barnard R. A. Wittenburg L. A. Amaravadi R. K. Gustafson D. L. Thorburn A. Thamm D. H. (2014). Phase I clinical trial and pharmacodynamic evaluation of combination hydroxychloroquine and doxorubicin treatment in pet dogs treated for spontaneously occurring lymphoma. Autophagy 10 (8), 1415–1425. 10.4161/auto.29165 24991836 PMC4203518

[B9] Booth L. A. Roberts J. L. Paul D. (2019). The role of cell signaling in the crosstalk between autophagy and apoptosis in the regulation of tumor cell survival in response to sorafenib and neratinib. Semin. Cancer Biol. 10.1016/j.semcancer.2019.10.013 PMC7167338 31644944

[B10] Bosnjak M. Ristic B. Arsikin K. Mircic A. Suzin-Zivkovic V. Perovic V. (2014). Inhibition of mTOR-dependent autophagy sensitizes leukemic cells to cytarabine-induced apoptotic death. PLoS One 9 (4), e94374. 10.1371/journal.pone.0094374 24714637 PMC3979773

[B11] Brana I. Ocana A. Chen E. X. Razak A. R. Haines C. Lee C. (2014). A phase I trial of pantoprazole in combination with doxorubicin in patients with advanced solid tumors: evaluation of pharmacokinetics of both drugs and tissue penetration of doxorubicin. Invest. New Drugs 32 (6), 1269–1277. 10.1007/s10637-014-0159-5 25213162

[B12] Buccarelli M. Marconi M. Pacioni S. De Pascalis I. D'Alessandris Q. G. Martini M A. B. (2018). Inhibition of autophagy increases susceptibility of glioblastoma stem cells to temozolomide by igniting ferroptosis. Cell Death Dis. 9 (8), 841. 10.1038/s41419-018-0864-7 30082680 PMC6079099

[B13] Carew J. S. Giles F. J. Nawrocki S. T. (2008). Histone deacetylase inhibitors: mechanisms of cell death and promise in combination cancer therapy. Cancer Lett. 269 (1), 7–17. 10.1016/j.canlet.2008.03.037 18462867

[B14] Chen L. H. Loong C. C. Su T. L. Lee Y. J. Chu P. M. Tsai M. L. (2011). Autophagy inhibition enhances apoptosis triggered by BO-1051, an N-mustard derivative, and involves the ATM signaling pathway. Biochem. Pharmacol. 81 (5), 594–605. 10.1016/j.bcp.2010.12.011 21184746

[B15] Chen Y. S. Song H. X. Lu Y. Li X. Chen T. Zhang Y. (2011). Autophagy inhibition contributes to radiation sensitization of esophageal squamous carcinoma cells. Dis. Esophagus 24, 437–443. 10.1111/j.1442-2050.2010.01156.x 21166739

[B16] Chen H. Luo Z. Sun W. Zhang C. Sun H. Zhao N. (2013). Low glucose promotes CD133mAb-elicited cell death via inhibition of autophagy in hepatocarcinoma cells. Cancer Lett. 336 (1), 204–212. 10.1016/j.canlet.2013.04.031 23652197

[B17] Chen L. Ye H. L. Zhang G. Yao W. M. Chen X. Z. Zhang F. C. (2014). Autophagy inhibition contributes to the synergistic interaction between EGCG and doxorubicin to kill the hepatoma Hep3B cells. PLoS One 9 (1), e85771. 10.1371/journal.pone.0085771 24465696 PMC3897495

[B18] Dalby K. N. Tekedereli I. Lopez-Berestein G. Ozpolat A. B. (2010). Targeting the prodeath and prosurvival functions of autophagy as novel therapeutic strategies in cancer. Autophagy 6 (3), 322–329. 10.4161/auto.6.3.11625 20224296 PMC2914492

[B19] Denton D. Kumar S. (2019). Autophagy-dependent cell death. Cell Death Differ. 26 (4), 605–616. 10.1038/s41418-018-0252-y 30568239 PMC6460387

[B20] Di Lernia G. Leone P. Solimando A. G. Buonavoglia A. Saltarella I. Ria R. (2020). Bortezomib Treatment Modulates Autophagy in Multiple Myeloma. J. Clin. Med. 9 (2), 552. 10.3390/jcm9020552 PMC7073518 32085480

[B21] Dickstein R. J. Nitti G. Dinney C. P. Davies B. R. Kamat A. M. McConkey D. J. (2012). Autophagy limits the cytotoxic effects of the AKT inhibitor AZ7328 in human bladder cancer cells. Cancer Biol. Ther. 13 (13), 1325–1338. 10.4161/cbt.21793 22895070 PMC3493441

[B22] Dragowska W. H. Weppler S. A. Wang J. C. Wong L. Y. Kapanen A. I. Rawji J. S. (2013). Induction of Autophagy Is an Early Response to Gefitinib and a Potential Therapeutic Target in Breast Cancer. PLoS One 8 (10), e76503. 10.1371/journal.pone.0076503 24146879 PMC3795739

[B23] Duffy A. Le J. Sausville E. Emadi A. (2015). Autophagy modulation: a target for cancer treatment development. Cancer Chemother. Pharmacol. 75, 439–447. 10.1007/s00280-014-2637-z 25422156

[B24] Fei Qu P. W. Zhang K. Shi Y. Li Y. Li C. Lu J. (2019). Manipulation of Mitophagy by “All-in-One” Nanosensitizer Augments Sonodynamic Glioma Therapy. Autophagy. 10.1080/15548627.2019.1687210 PMC7480814 31674265

[B25] Feng Y. Gao Y. Wang D. Xu Z. Sun W. R P. (2018). Autophagy Inhibitor (LY294002) and 5-fluorouracil (5-FU) Combination-Based Nanoliposome for Enhanced Efficacy Against Esophageal Squamous Cell Carcinom. Nanoscale Res. Lett. 13 (1), 325. 10.1186/s11671-018-2716-x 30328537 PMC6192941

[B26] Fukuda T. Oda K. Wada-Hiraike O. Sone K. Inaba K. Ikeda Y. (2015). The anti-malarial chloroquine suppresses proliferation and overcomes cisplatin resistance of endometrial cancer cells via autophagy inhibition. Gynecol. Oncol. 137 (3), 538–545. 10.1016/j.ygyno.2015.03.053 25842161

[B27] Fulda S. Kögel D. (2015). Cell death by autophagy: emerging molecular mechanisms and implications for cancer therapy. Oncogene 34 (40), 5105–5113. 10.1038/onc.2014.458 25619832

[B28] G D. A. (2014). The four faces of autophagy: implications for cancer therapy. Cancer Res. 73 (3), 647–651. 10.1158/0008-5472.CAN-13-2966 24459182

[B29] Galati S. Boni C. Gerra M. C. Lazzaretti M. Buschini A. (2019). Autophagy. A Player in response to Oxidative Stress and DNA Damage. Oxid. Med. Cell. Longevity 2019, 5692958. 10.1155/2019/5692958 PMC6701339 31467633

[B30] Ganesher A. Chaturvedi P. Sahai R. Meena S. Mitra K. Datta D. (2020). New Spisulosine Derivative promotes robust autophagic response to cancer cells. Eur. J. Med. Chem. 188, 112011. 10.1016/j.ejmech.2019.112011 31926468

[B31] Gewirtz D. A. (2014). An autophagic switch in the response of tumor cells to radiation and chemotherapy. Biochem. Pharmacol. 90, 208–211. 10.1016/j.bcp.2014.05.016 24875447

[B32] Giulia Petroni G. B. Iorio J. Duranti C. Lottini T. Stefanini M. Kragol G. (2020). Clarithromycin inhibits autophagy in colorectal cancer by regulating the hERG1 potassium channel interaction with PI3K. Cell Death Dis. 11, 161. 10.1038/s41419-020-2349-8 32123164 PMC7052256

[B33] Golden E. B. Cho H. Y. Hofman F. M. Louie S. G. Schonthal A. H. Chen T. C. (2015). Quinoline-based antimalarial drugs: a novel class of autophagy inhibitors. Neurosurg. Focus 38 (3), E12. 10.3171/2014.12.FOCUS14748 25727221

[B34] Gong F. Peng X. Sang Y. Qiu M. Luo C. He Z. (2013). Dichloroacetate induces protective autophagy in LoVo cells: involvement of cathepsin D/thioredoxin-like protein 1 and Akt-mTOR-mediated signaling. Cell Death Dis. 7 (4), e913. 10.1038/cddis.2013.438 PMC3847316 24201812

[B35] Grimaldi A. Santini D. Zappavigna S. Lombardi A. Misso G. Boccellino M. (2015). Antagonistic effects of chloroquine on autophagy occurrence potentiate the anticancer effects of everolimus on renal cancer cells. Cancer Biol. Ther. 16 (4), 567–579. 10.1080/15384047.2015.1018494 25866016 PMC4622435

[B36] Guo X.-l. Li D. Sun K. Wang J. Liu Y. Song J.-r. (2013). Inhibition of autophagy enhances anticancer effects of bevacizumab in hepatocarcinoma. J. Mol. Med. 91, 473–483. 10.1007/s00109-012-0966-0 23052483 PMC3611041

[B37] Guo Y. Huang C. Li G. Chen T. Li J. Huang Z. (2015). Paxilitaxel induces apoptosis accompanied by protective autophagy in osteosarcoma cells through hypoxia-inducible factor-1alpha pathway. Mol. Med. Rep. 12 (3), 3681–3687. 10.3892/mmr.2015.3860 26017247

[B38] Haiyang Yu C.-L. W. Wang X. Ban Q. Quan C. Liu M. Dong H. (2019). SP600125 enhances C-2-induced cell death by the switch from autophagy to apoptosis in bladder cancer cells. J. Exp. Clin. Cancer Res. 2019 (38), 448. 10.1186/s13046-019-1467-6 PMC6829950 31685029

[B39] Hong Z. F. Zhao W. X. Yin Z. Y. Xie C. R. Xu Y. P. Chi X. Q. (2015). Capsaicin Enhances the Drug Sensitivity of Cholangiocarcinoma through the Inhibition of Chemotherapeutic-Induced Autophagy. PLoS One 10 (5), e0121538. 10.1371/journal.pone.0121538 25933112 PMC4416771

[B40] Honma Y. Harada M. (2013). Sorafenib enhances proteasome inhibitor-mediated cytotoxicity via inhibition of unfolded protein response and keratin phosphorylation. Exp. Cell Res. 319 (14), 2166–2167. 10.1016/j.yexcr.2013.05.023 23727131

[B41] Horwacik I. Gaik M. Durbas M. Boratyn E. Zajac G. Szychowska K. (2015). Inhibition of autophagy by 3-methyladenine potentiates sulforaphane-induced cell death of BE(2)-C human neuroblastoma cells. Mol. Med. Rep. 12 (1), 535–542. 10.3892/mmr.2015.3377 25695841

[B42] Hsieh M. J. Lin C. W. Yang S. F. Sheu G. T. Yu Y. Y. Chen M. K. (2013). A Combination of Pterostilbene With Autophagy Inhibitors Exerts Efficient Apoptotic Characteristics in Both Chemosensitive and Chemoresistant Lung Cancer Cells. Toxicol. Sci. 137 (1), 65–75. 10.1093/toxsci/kft238 24154491

[B43] Hu T. Li Z. Gao C. Y. Cho C. H. (2016). Mechanisms of drug resistance in colon cancer and its therapeutic strategies. World J. Gastroenterol. 22 (30), 6876–6889. 10.3748/wjg.v22.i30.6876 27570424 PMC4974586

[B44] Huang S. Sinicrope F. A. (2010). Celecoxib-induced apoptosis is enhanced by ABT-737 and by inhibition of autophagy in human colorectal cancer cells. Autophagy 6 (2), 256–269. 10.4161/auto.6.2.11124 20104024 PMC2948490

[B45] Jarauta V. Jaime P. Gonzalo O. de Miguel D. Ramirez-Labrada A. Martinez-Lostao L. (2016). Inhibition of autophagy with chloroquine potentiates carfilzomib-induced apoptosis in myeloma cells in vitro and in vivo. Cancer Lett. 382 (1), 1–10. 10.1016/j.canlet.2016.08.019 27565383

[B46] Jiang H. Wang H. Zou W. Hu Y. Chen C. Wang C. (2019). Sufentanil impairs autophagic degradation and inhibits cell migration in NCI-H460. Oncol. Lett. 18 (6), 6829–6835. 10.3892/ol.2019.10997 31788126 PMC6865617

[B47] Jiménez-Guerrero R. Gasca J. Flores M. L. Pérez-Valderrama B. Tejera-Parrado C. Medina R. (2018). Obatoclax and Paclitaxel Synergistically Induce Apoptosis and Overcome Paclitaxel Resistance in Urothelial Cancer Cells. Cancers (Basel) 10 (12), E490. 10.3390/cancers10120490 30563080 PMC6316685

[B48] Jing Z. Sui X. Yao J. Xie J. Jiang L. Zhou Y. (2016). SKF-96365 activates cytoprotective autophagy to delay apoptosis in colorectal cancer cells through inhibition of the calcium/CaMKIIγ/AKT-mediated pathway. Cancer Lett. 372 (2), 226–238. 10.1016/j.canlet.2016.01.006 26803057 PMC5240807

[B49] Jingdong Rao L. Liu J. Tang X. Yin S. Xia C. Wei J. (2019). Size-adjustable micelles co-loaded with a chemotherapeutic agent and an autophagy inhibitor for enhancing cancer treatment via increased tumor retention. Acta Biomater. 89, 300–312. 10.1016/j.actbio.2019.03.022 30878446

[B50] Junco J. J. Mancha-Ramirez A. Malik G. Wei S. J. Kim D. J. Liang H. (2015). Ursolic acid and resveratrol synergize with chloroquine to reduce melanoma cell viability. Melanoma Res. 25 (2), 103–112. 10.1097/CMR.0000000000000137 25647735

[B51] Jung H.-J. Seo I. Casciello F. Jacquelin S. Lane S. W. S-Il Suh M.-H. S. (2016). The anticancer effect of chaetocin is enhanced by inhibition of autophagy. Cell Death Dis. 7, e2098. 10.1038/cddis.2016.15 26890137 PMC5399187

[B52] Kimura T. Takabatake Y. Takahashi A. Isaka A. Y. (2012). Chloroquine in Cancer Therapy: A Double-Edged Sword of Autophagy. Cancer Res. 7 (31), 3–7. 10.1158/0008-5472.CAN-12-2464 23288916

[B53] Kiruthiga C. Devi K. P. Nabavi S. M. Autophagy B. A. (2020). A Potential Therapeutic Target of Polyphenols in Hepatocellular Carcinoma. Cancers (Basel) 12 (3), E562. 10.3390/cancers12030562 32121322 PMC7139730

[B54] Kleger A. Perkhofer L. Seufferlein T. (2014). Smarter drugs emerging in pancreatic cancer therapy. Ann. Oncol. 25 (7), 1260–1270. 10.1093/annonc/mdu013 24631947

[B55] Koehler B. C. Jassowicz A. Scherr A. L. Lorenz S. Radhakrishnan P. Kautz N. (2015). Pan-Bcl-2 inhibitor Obatoclax is a potent late stage autophagy inhibitor in colorectal cancer cells independent of canonical autophagy signaling. BMC Cancer 15, 919. 10.1186/s12885-015-1929-y 26585594 PMC4653869

[B56] Kumar A. Singh U. K. Chaudhary A. (2015). Targeting autophagy to overcome drug resistance in cancer therapy. Future Med. Chem. 7 (12), 1535–1542. 10.4155/fmc.15.88 26334206

[B57] Kuroda J. Shimura Y. Yamamoto - Sugitani M. Taniwaki N. S. A. M. (2013). Multifaceted Mechanisms for Cell Survival and Drug Targeting in Chronic Myelogenous Leukemia. Curr. Cancer Drug Targets 13, 69–79. 10.2174/156800913804486638 22414011

[B58] Lalita Guntuku J. K. G. Thummuri D. Borkar R. M. Manavathi B. Ragampeta S. Vaidya J. R. (2019). IITZ-01, a novel potent lysosomotropic autophagy inhibitor, has single-agent antitumor efficacy in triple-negative breast cancer in vitro and in vivo. Oncogene 38 (4) 581–595. 10.1038/s41388-018-0446-2 30166591

[B59] Lamoureux F. Thomas C. Crafter C. Kumano M. Zhang F. Davies B. R. (2013). Blocked autophagy using lysosomotropic agents sensitizes resistant prostate tumor cells to the novel Akt inhibitor AZD5363. Clin. Cancer Res. 19 (4), 833–844. 10.1158/1078-0432.CCR-12-3114 23258740

[B60] Law B. Y. K. Mok S. W. F. Chen J. Michelangeli F. Jiang Z. H. Han Y. (2017). N-Desmethyldauricine Induces Autophagic Cell Death in Apoptosis-Defective Cells via Ca Mobilization. Front. Pharmacol. 8, 388. 10.3389/fphar.2017.00388 28670281 PMC5472688

[B61] Li J. Hou N. Faried A. Tsutsumi S. Kuwano H. (2010). Inhibition of autophagy augments 5-fluorouracil chemotherapy in human colon cancer in vitro and in vivo model. Eur. J. Cancer 46 (10), 1900–1909. 10.1016/j.ejca.2010.02.021 20231086

[B62] Li Y. Luo P. Wang J. Dai J. Yang X. Wu H. (2014). Autophagy blockade sensitizes the anticancer activity of CA-4 via JNK-Bcl-2 pathway. Toxicol. Appl. Pharmacol. 274 (2), 319–327. 10.1016/j.taap.2013.11.018 24321340

[B63] Li K. Chen X. Liu C. Gu P. Li Z. Wu S. (2015). Pirarubicin induces an autophagic cytoprotective response through suppression of the mammalian target of rapamycin signaling pathway in human bladder cancer cells. Biochem. Biophys. Res. Commun. 460 (2), 380–385. 10.1016/j.bbrc.2015.03.042 25791481

[B64] Li C. Liu Y. Liu H. Zhang W. Shen C. Cho K. (2015). Impact of autophagy inhibition at different stages on cytotoxic effect of autophagy inducer in glioblastoma cells. Cell Physiol. Biochem. 35 (4), 1303–1316. 10.1159/000373952 25721868

[B65] Li Y. McGreal S. Zhao J. Huang R. Zhou Y. Zhong H. (2016). A cell-based quantitative high-throughput image screening identified novel autophagy modulators. Pharmacol. Res. 110, 35–49. 10.1016/j.phrs.2016.05.004 27168224 PMC4995889

[B66] Liang X. De Vera M. E. Buchser W. J. de Vivar Chavez A. R. Loughran P. Stolz D. B. (2012). Inhibiting Autophagy During Interleukin 2 (IL-2) Immunotherapy Promotes Long Term Tumor Regression. Cancer Res. 2 (11), 2791–2801. 10.1158/0008-5472.CAN-12-0320 PMC3417121 22472122

[B67] Lin C. J. Lee C. C. Shih Y. L. Lin T. Y. Wang S. H. Lin Y. F. (2012). Resveratrol enhances the therapeutic effect of temozolomide against malignant glioma in vitro and in vivo by inhibiting autophagy. Free Radic. Biol. Med. 52 (2), 377–391. 10.1016/j.freeradbiomed.2011.10.487 22094224

[B68] Liu L. Yang M. Kang R. Wang Z. Zhao Y. Yu Y. (2011). DAMP-mediated autophagy contributes to drug resistance. Autophagy 7 (1), 112–114. 10.4161/auto.7.1.14005 21068541 PMC3039734

[B69] Liu W. Zhang Z. Zhang Y. Chen X. Guo S. Lei Y. (2015). HMGB1-mediated autophagy modulates sensitivity of colorectal cancer cells to oxaliplatin via MEK/ERK signaling pathway. Cancer Biol. Ther. 16 (4), 511–517. 10.1080/15384047.2015.1017691 25778491 PMC4622507

[B70] Liu J. T. Li W. C. Gao S. Wang F. Li X. Q. Yu H. Q. (2015). Autophagy Inhibition Overcomes the Antagonistic Effect Between Gefitinib and Cisplatin in Epidermal Growth Factor Receptor Mutant Non–Small-Cell Lung Cancer Cells. Clin. Lung Cancer 15 (5), e55–e66. 10.1016/j.cllc.2015.03.006 25979647

[B71] Liu X. Sun K. I. Wang H. Dai Y. (2016). Inhibition of Autophagy by Chloroquine Enhances the Antitumor Efficacy of Sorafenib in Glioblastoma. Cell Mol. Neurobiol. 36, 1197–1208. 10.1007/s10571-015-0318-z 26971793 PMC11482299

[B72] Liu M. Bamodu O. A. Huang W. C. Zucha M. A. Lin Y. K. Wu A. T. H. (2017). 4-Acetylantroquinonol B suppresses autophagic flux and improves cisplatin sensitivity in highly aggressive epithelial cancer through the PI3K/Akt/mTOR/p70S6K signaling pathway. Toxicol. Appl. Pharmacol. 325, 48–60. 10.1016/j.taap.2017.04.003 28408137

[B73] Liu Z. He K. Ma Q. Yu Q. Liu C. Ndege I. (2017). Autophagy inhibitor facilitates gefitinib sensitivity in vitro and in vivo byactivating mitochondrialapoptosis in triple negative breast cancer. PloS One 12 (5), e0177694. 10.1371/journal.pone.0177694 28531218 PMC5439698

[B74] Liu H. Sun T. Ming L. (2017). Multiple Roles of Autophagy in the Sorafenib Resistance of Hepatocellular Carcinoma. Cell. Physiol. Biochem. 44 (2), 716–727. 10.1159/000485285 29169150

[B75] Lobo M. R. Green S. C. Schabel M. C. Gillespie G. Y. Woltjer R. L. Pike M. M. (2013). Quinacrine synergistically enhances the antivascular and antitumor efficacy of cediranib in intracranial mouse glioma. Neuro Oncol. 15 (12), 1673–1683. 10.1093/neuonc/not119 24092859 PMC3829589

[B76] Lv X. Liu F. Shang Y. Chen S. Z. (2015). Honokiol exhibits enhanced antitumor effects with chloroquine by inducing cell death and inhibiting autophagy in human non-small cell lung cancer cells. Oncol. Rep. 34 (3), 1289–1300. 10.3892/or.2015.4091 26136140

[B77] Mahalingam D. Mita M. Sarantopoulos J. Wood L. Amaravadi R. K. Davis L. E. (2014). Combined autophagy and HDAC inhibition: a phase I safety, tolerability, pharmacokinetic, and pharmacodynamic analysis of hydroxychloroquine in combination with the HDAC inhibitor vorinostat in patients with advanced solid tumors. Autophagy 10 (8), 1403–1414. 10.4161/auto.29231 24991835 PMC4203517

[B78] Mahoney E. Lucas D. M. Gupta S. V. Wagner A. J. Herman S. E. Smith L. L. (2012). ER stress and autophagy: new discoveries in the mechanism of action and drug resistance of the cyclin-dependent kinase inhibitor flavopiridol. Blood 120 (6), 1262–1273. 10.1182/blood-2011-12-400184 22740450 PMC3418721

[B79] Mei L. Chen Y. Wang Z. Wang J. Wan J. Yu C. (2015). Synergistic anti-tumour effects of tetrandrine and chloroquine combination therapy in human cancer: a potential antagonistic role for p21. Br. J. Pharmacol. 172 (9), 2232–2245. 10.1111/bph.13045 25521075 PMC4403090

[B80] Mei-Chuan Chen Y.-C. L. Liao Y.-H. Liou J.-P. Chen C.-H. (2019). MPT0G612, a Novel HDAC6 Inhibitor, Induces Apoptosis and Suppresses IFN-γ-Induced Programmed Death-Ligand 1 in Human Colorectal Carcinoma Cells. Cancers 2019 (11), 1617. 10.3390/cancers11101617 PMC6826904 31652644

[B81] Meng J. Chang1 C. Chen Y. Bi F. Ji C. Liu W. (2019). EGCG overcomes gefitinib resistance by inhibiting autophagy and augmenting cell death through targeting ERK phosphorylation in NSCLC. Onco. Targets Ther. 12, 6033–6043. 10.2147/OTT.S209441 31440060 PMC6668247

[B82] Misirkic M. Janjetovic K. Vucicevic L. Tovilovic G. Ristic B. Vilimanovich U. (2012). Inhibition of AMPK-dependent autophagy enhances in vitro antiglioma effect of simvastatin. Pharmacol. Res. 65 (1), 111–119. 10.1016/j.phrs.2011.08.003 21871960

[B83] Miyazawa K. (2011). Combined treatment with bortezomib plus bafilomycin A1 enhances the cytocidal effect and induces endoplasmic reticulum stress in U266 myeloma cells: Crosstalk among proteasome, autophagy-lysosome and ER stress. Int. J. Oncol. 38 (3), 643–654. 10.3892/ijo.2010.882 21174067

[B84] Moriya S. Che X. F. Komatsu S. Abe A. Kawaguchi T. Gotoh A. (2013). Macrolide antibiotics block autophagy flux and sensitize to bortezomib via endoplasmic reticulum stress-mediated CHOP induction in myeloma cells. Int. J. Oncol. 42 (5), 1541–1550. 10.3892/ijo.2013.1870 23546223 PMC3661227

[B85] Nagelkerke A. Bussink J. Geurts-Moespot A. Fred C. G. J. S. Span P. N. (2015). Therapeutic targeting of autophagy in cancer. Part II: Pharmacological modulation of treatment-induced autophagy. Semin. Cancer Biol. 31, 99–105. 10.1016/j.semcancer.2014.06.001 24933034

[B86] Natsumeda M. Maitani K. Liu Y. Miyahara H. Kaur H. Chu Q. (2016). Targeting Notch Signaling and Autophagy Increases Cytotoxicity in Glioblastoma Neurospheres. Brain Pathol. 26 (6), 713–723. 10.1111/bpa.12343 26613556 PMC8029219

[B87] Ojha R. Singh S. K. Bhattacharyya S. (2016). JAK-mediated autophagy regulates stemness and cell survival in cisplatin resistant bladder cancer cells. Biochim. Biophys. Acta 1860 (11 Pt A), 2484–2497. 10.1016/j.bbagen.2016.07.021 27474203

[B88] Pan B. Chen D. Huang J. Wang R. Feng B. H1 S. (2014). HMGB1-mediated autophagy promotes docetaxel resistance in human lung adenocarcinoma. Mol. Cancer Ther. 13, 165. 10.1186/1476-4598-13-165 24996221 PMC4125709

[B89] Pan H. Wang Z. Jiang L. Sui X. You L. Shou J. (2014). Autophagy inhibition sensitizes hepatocellular carcinoma to the multikinase inhibitor linifanib. Sci. Rep. 4, 6683. 10.1038/srep06683 25327881 PMC4202209

[B90] Pan Y. Z. Wang X. Bai H. Wang C. B. Zhang Q. Xi R. (2015). Autophagy in drug resistance of the multiple myeloma cell line RPMI8226 to doxorubicin. Genet. Mol. Res. 14 (2), 5621–5629. 10.4238/2015.May.25.14 26125760

[B91] Pei-Feng Liu1 K.-L. T. Hsu C.-J. Tsai W.-L. Cheng J.-S. Chang H.-W. Shiau C.-W. (2018) Drug Repurposing Screening Identifies Tioconazole as an ATG4 Inhibitor that Suppresses Autophagy and Sensitizes Cancer Cells to Chemotherapy. Theranostics 8 (3) 830–845. 10.7150/thno.22012 29344310 PMC5771097

[B92] Pellerito C. Emanuele S. Ferrante F. Celesia A. Giuliano M. Tiziana F. (2020). Tributyltin(IV) ferulate, a novel synthetic ferulic acid derivative, induces autophagic cell death in colon cancer cells: From chemical synthesis to biochemical effects. J. Inorg. Biochem. 205, 110999. 10.1016/j.jinorgbio.2020.110999 31986423

[B93] Peng X. Gong F. Chen Y. Jiang Y. Liu J. Yu M. (2014). Autophagy promotes paclitaxel resistance of cervical cancer cells: involvement of Warburg effect activated hypoxia-induced factor 1-alpha-mediated signaling. Cell Death Dis. 5, e1367. 10.1038/cddis.2014.297 25118927 PMC4454295

[B94] Pengxuan Zhao M. L. Wang Y. Chen Y. He C. Zhang X. Yang T. (2018). Enhancing anti-tumor efficiency in hepatocellular carcinoma through the autophagy inhibition by miR-375/sorafenib in lipid-coated calcium carbonate nanoparticles. Acta Biomater. 05 (72), 248–255. 10.1016/j.actbio.2018.03.022 29555460

[B95] Poklepovic A. Gewirtz D. A. (2014). Outcome of early clinical trials of the combination of hydroxychloroquine with chemotherapy in cancer. Autophagy 10 (8), 1478–1480. 10.4161/auto.29428 24991829 PMC4203528

[B96] Qianhua Feng X. Y. Hao Y. Wang N. Feng X. Hou L. Zhang Z. (2019). Cancer Cell Membrane-Biomimetic Nanoplatform for Enhanced Sonodynamic Therapy on Breast Cancer via Autophagy Regulation Strategy. ACS Appl. Mater. Interf. 11, 32729–32738. 10.1021/acsami.9b10948 31415145

[B97] Rai G. Mishra S. Suman S. Shukla Y. (2016). Resveratrol improves the anticancer effects of doxorubicin in vitro and in vivo models: A mechanistic insight. Phytomedicine 23 (3), 233–242. 10.1016/j.phymed.2015.12.020 26969377

[B98] Rangwala R. Leone R. Chang Y. C. Fecher L. A. Schuchter L. M. Kramer A. (2014). Phase I trial of hydroxychloroquine with dose-intense temozolomide in patients with advanced solid tumors and melanoma. Autophagy 10 (8), 1369–1379. 10.4161/auto.29118 24991839 PMC4203514

[B99] Rangwala R. Chang Y. C. Hu J. Algazy K. M. Evans T. L. Fecher L. A. (2014). Combined MTOR and autophagy inhibition: phase I trial of hydroxychloroquine and temsirolimus in patients with advanced solid tumors and melanoma. Autophagy 10 (8), 1391–1402. 10.4161/auto.29119 24991838 PMC4203516

[B100] Rosenfeld M. R. Ye X. Supko J. G. Desideri S. Grossman S. A. Brem S. (2014). A phase I/II trial of hydroxychloroquine in conjunction with radiation therapy and concurrent and adjuvant temozolomide in patients with newly diagnosed glioblastoma multiforme. Autophagy 10 (8), 1359–1368. 10.4161/auto.28984 24991840 PMC4203513

[B101] Saavedra-García P. Martini F. HW A. (2020). Proteasome inhibition in multiple myeloma: lessons for other cancers. Am. J. Physiol. Cell Physiol. 318 (3), C451–C462. 10.1152/ajpcell.00286.2019 31875696

[B102] Sannigrahi M. K. Singh V. Sharma R. Panda N. K. Khullar M. (2015). Role of autophagy in head and neck cancer and therapeutic resistance. Oral Dis. 21 (3), 283–291. 10.1111/odi.12254 24797102

[B103] Sasaki K. Tsuno N. H. Sunami E. Tsurita G. Kawai K. Okaji Y. (2010). Chloroquine potentiates the anti-cancer effect of 5-fluorouracil on colon cancer cells. BMC Cancer 10, 370. 10.1186/1471-2407-10-370 20630104 PMC2914703

[B104] Shen W. Zhang X. Fu X. Fan J. Luan J. Cao Z. (2017). A novel and promising therapeutic approach for NSCLC: recombinant human arginase alone or combined with autophagy inhibitor. Cell Death Dis. 8 (3), e2720. 10.1038/cddis.2017.137 28358368 PMC5386540

[B105] Sheng Yin C. X. Wang Y. Wan D. Rao J. Tang X. Wei J. (2018). Dual receptor recognizing liposomes containing paclitaxel and hydroxychloroquine for primary and metastatic melanoma treatment via autophagy-dependent and independent pathways. J. Control Release 10 (28), 288. 10.1016/j.jconrel.2018.08.015 30099017

[B106] Shi Y. M. Yang L. Geng Y. D. Zhang C. Kong L. Y. (2015). Polyphyllin I induced-apoptosis is enhanced by inhibition of autophagy in human hepatocellular carcinoma cells. Phytomedicine 22 (13), 1139–1149. 10.1016/j.phymed.2015.08.014 26598912

[B107] Shin S. W. Kim S. Y. Park J. W. (2012). Autophagy inhibition enhances ursolic acid-induced apoptosis in PC3 cells. Biochim. Biophys. Acta 1823 (2), 451–457. 10.1016/j.bbamcr.2011.10.014 22178132

[B108] Shingu T. Fujiwara K. Bogler O. Akiyama Y. Moritake K. Shinojima N. (2009). Inhibition of autophagy at a late stage enhances imatinib-induced cytotoxicity in human malignant glioma cells. Int. J. Cancer 124 (5), 1060–1071. 10.1002/ijc.24030 19048625

[B109] Simonet S. Rodriguez-Lafrasse C. Beal D. Gerbaud S. Malesys C. Tillement O. (2020). Gadolinium-Based Nanoparticles Can Overcome the Radioresistance of Head and Neck Squamous Cell Carcinoma Through the Induction of Autophagy. J. BioMed. Nanotechnol. 16 (1) 111–124. 10.1166/jbn.2020.2871 31996290

[B110] Song P. Li Y. Dong Y. Liang Y. Qu H. Qi D. (2019). Estrogen receptor β inhibits breast cancer cells migration and invasion through CLDN6-mediated autophagy. J. Exp. Clin. Cancer Res. 38 (1), 354. 10.1186/s13046-019-1359-9 31412908 PMC6694553

[B111] Stanton M. J. Dutta S. Zhang H. Polavaram N. S. Leontovich A. A. Hönscheid P. (2013). Autophagy control by the VEGF-C/NRP-2 axis in cancer and its implication for treatment resistance. Cancer Res. 73 (1), 160–171. 10.1158/0008-5472.CAN-11-3635 23149913 PMC3805049

[B112] Sugita S. Ito K. Yamashiro Y. Moriya S. Che X.-F. Yokoyama T. (2015). EGFR-independent autophagy induction with gefitinib and enhancemnt of its cytotoxic effect by targeting autophagy with clarithromycin in non -small cell lung cancer cells. Biochem. Biophys. Res. Commun. 461, 28–34. 10.1016/j.bbrc.2015.03.162 25858318

[B113] Tan Q. Wang H. Hu Y. Hu M. Li X. Ma A. Y. (2015). Src/STAT3-dependent heme oxygenase-1 induction mediates chemoresistance of breast cancer cells to doxorubicin by promoting autophagy. Cancer Sci. 106 (8), 1023–1032. 10.1111/cas.12712 26041409 PMC4556392

[B114] Thorburn A. Thamm D. H. Gustafson D. L. (2014). Autophagy and cancer therapy. Mol. Pharmacol. 85 (6), 830–838. 10.1124/mol.114.091850 24574520 PMC4014668

[B115] Townsend K. N. Hughson L. R. K. Schlie K. Poon V. I. Westerback A. Lum J. J. (2012). Autophagy inhibition in cancer therapy: metabolic considerations for antitumor immunity. Immunol. Rev. 249, 176–194. 10.1111/j.1600-065X.2012.01141.x 22889222

[B116] Vilimanovich U. Bosnjak M. Bogdanovic A. Markovic I. Isakovic A. Kravic-Stevovic T. (2015). Statin-mediated inhibition of cholesterol synthesis induces cytoprotective autophagy in human leukemic cells. Eur. J. Pharmacol. 765, 415–428. 10.1016/j.ejphar.2015.09.004 26358205

[B117] Vogl D. T. Stadtmauer E. A. Tan K. S. Heitjan D. F. Davis L. E. Pontiggia L. (2014). Combined autophagy and proteasome inhibition: a phase 1 trial of hydroxychloroquine and bortezomib in patients with relapsed/refractory myeloma. Autophagy 10 (8), 1380–1390. 10.4161/auto.29264 24991834 PMC4203515

[B118] Wang W. Ding Chen K. Z. (2018). SOX2OT variant 7 contributes to the synergistic interaction between EGCG and Doxorubicin to kill osteosarcoma via autophagy and stemness inhibition. J. Exp. Clin. Cancer Res. 37 (1), 37. 10.1186/s13046-018-0689-3 29475441 PMC6389193

[B119] Wang J. GS W. (2014). Role of autophagy in cisplatin resistance in ovarian cancer cells. J. Biol. Chem. 289 (24), 17163–17173. 10.1074/jbc.M114.558288 24794870 PMC4059157

[B120] Wang L. Zhang H. Sun M. Yin Z. Qian J. (2015). High mobility group box 1-mediated autophagy promotes neuroblastoma cell chemoresistance. Oncol. Rep. 34 (6), 2969–2976. 10.3892/or.2015.4278 26397184

[B121] Wang L. Zhu Y.-R. Wang S. Zhao S. (2016). Autophagy inhibition sensitizes WYE-354-induced anti-colon cancer activity in vitro and in vivo. Tumor Biol. 37, 11743–11752. 10.1007/s13277-016-5018-x 27020593

[B122] Wang D. Guo H. Yang H. Wang D. Gao P. Wei W. (2019). Pterostilbene, An Active Constituent of Blueberries, Suppresses Proliferation Potential of Human Cholangiocarcinoma Enhancing the Autophagic Flux. Front. Pharmacol. 10, 1238. 10.3389/fphar.2019.01238 31695612 PMC6817474

[B123] Wang Y P. R. Li D. D. Ding Y. Wu X. Q. Zeng Y. X. Zhu X. F. (2011). Chloroquine enhances the cytotoxicity of topotecan by inhibiting autophagy in lung cancer cells. Chin. J. Cancer 30 (10), 690–700. 10.5732/cjc.011.10056 21959046 PMC4012269

[B124] Wei-Chih Chen K.-Y. H. Hung C.-M. Lin Y.-C. Ning-Sun Yang C.-T. H. Kuog S.-C. Way T.-D. (2014). The anti-tumor efficiency of pterostilbene is promoted with a combined treatment of Fas signaling or autophagy inhibitors in triple negative breast cancer cells. Food Funct. 5, 1856. 10.1039/C4FO00145A 24944076

[B125] White E. Mehnert J. M. Chang C. S. (2015). Autophagy, Metabolism, and Cancer. Clin. Cancer Res. 21 (22), 5037–5046. 10.1158/1078-0432.CCR-15-0490 26567363 PMC4646728

[B126] White E. (2015). The role for autophagy in cancer. J. Clin. Invest. 125 (1), 42–46. 10.1172/JCI73941 25654549 PMC4382247

[B127] Wolpin B. M. Rubinson D. A. Wang X. Chan J. A. Cleary J. M. Enzinger P. C. (2014). Phase II and pharmacodynamic study of autophagy inhibition using hydroxychloroquine in patients with metastatic pancreatic adenocarcinoma. Oncologist 19 (6), 637–638. 10.1634/theoncologist.2014-0086 24821822 PMC4041680

[B128] Wu T. Wang M. C. Jing L. Liu Z. Y. Guo H. Liu Y. (2015). Autophagy facilitates lung adenocarcinoma resistance to cisplatin treatment by activation of AMPK/mTOR signaling pathway. Drug Des. Dev. Ther. 9, 6421–6431. 10.2147/DDDT.S95606 PMC4686226 26715839

[B129] Xu K. Park D. Magis A. T. Zhang J. Zhou W. Sica G. L. (2019). Small Molecule KRAS Agonist for Mutant KRAS Cancer Therapy. Mol. Cancer 18 (1), 85. 10.1186/s12943-019-1012-4 30971271 PMC6456974

[B130] Yan Y. Xu Z. Dai S. Qian L. Sun L. Gong Z. (2016). Targeting autophagy to sensitive glioma to temozolomide treatment. J. Exp. Clin. Cancer Res. 35, 23. 10.1186/s13046-016-0303-5 26830677 PMC4736617

[B131] Yang Z. J. Chee C. E. Huang S. Sinicrope F. A. (2011). The role of autophagy in cancer: therapeutic implications. Mol. Cancer Ther. 10 (9), 1533–1541. 10.1158/1535-7163.MCT-11-0047 21878654 PMC3170456

[B132] Yang Y. P. Hu L. F. Zheng H. F. Mao C. J. Hu W. D. Xiong K. P. (2013). Application and interpretation of current autophagy inhibitors and activators. Acta Pharmacol. Sin. 34 (5), 625–635. 10.1038/aps.2013.5 23524572 PMC4002883

[B133] Yang L. Wan J. Xiao S. Barkhouse D. Zhu J. Li G. (2016). BH3 mimetic ABT-737 sensitizes colorectal cancer cells to ixazomib through MCL-1 downregulation and autophagy inhibition. Am. J. Cancer Res. 2, 6 (6), 1345–1357. 10.1016/j.ijrobp.2016.06.2028 PMC4937737 27429848

[B134] Ye H. Chen M. Cao F. Huang H. Zhan R. Zheng A. X. (2016). Chloroquine, an autophagy inhibitor, potentiates the radiosensitivity of glioma initiating cells by inhibiting autophagy and activating apoptosis. BMC Neurol. 16, 178. 10.1186/s12883-016-0700-6 27644442 PMC5029068

[B135] Yin X. Zhang N. Di W. (2013). Regulation of LC3-dependent protective autophagy in ovarian cancer cells by protein phosphatase 2A. Int. J. Gynecol. Cancer 23 (4), 630–641. 10.1097/IGC.0b013e3182892cee 23518861

[B136] Yongxi T. Haijun H. Jiaping Z. Guoliang S. Hongying P. (2015). Autophagy inhibition sensitizes KU-0063794-mediated anti-HepG2 hepatocellular carcinoma cell activity in vitro and in vivo. Biochem. Biophys. Res. Commun. 465, 494–500. 10.1016/j.bbrc.2015.08.045 26278819

[B137] Yu L. Gu C. Zhong D. Shi L. Kong Y. Zhou Z. (2014). Induction of autophagy counteracts the anticancer effect of cisplatin in human esophageal cancer cells with acquired drug resistance. Cancer Lett. 355 (1), 34–45. 10.1016/j.canlet.2014.09.020 25236911

[B138] Yuan G. Yan S. F. Xue H. Zhang P. Sun J. T. Li G. (2014). Cucurbitacin I induces protective autophagy in glioblastoma in vitro and in vivo. J. Biol. Chem. 289 (15), 10607–10619. 10.1074/jbc.M113.528760 24599950 PMC4036180

[B139] Yuan H. Li A.-J. Ma S.-L. Cui L.-J. Bin Wu L. Y. Wu M.-C. (2014). Inhibition of autophagy significantly enhances combination therapy with sorafenib and HDAC inhibitors for human hepatoma cells. World J. Gastroenterol. 20 (17), 4953–4962. 10.3748/wjg.v20.i17.4953 24833845 PMC4009527

[B140] Zang Y. Thomas S. M. Chan E. T. Kirk C. J. Freilino M. L. DeLancey H. M. (2012). The next generation proteasome inhibitors carfilzomib and oprozomib activate prosurvival autophagy via induction of the unfolded protein response and ATF4. Autophagy 8 (12), 1873–1874. 10.4161/auto.22185 22995770 PMC3541310

[B141] Zeng X. Zhao H. Li Y. Fan J. Sun Y. Wang S. (2015). Targeting Hedgehog signaling pathway and autophagy overcomes drug resistance of BCR-ABL-positive chronic myeloid leukemia. Autophagy 11 (2), 355–372. 10.4161/15548627.2014.994368 25701353 PMC4502679

[B142] Zhan Z. Li Q. Wu P. Ye Y. Tseng H. Y. Zhang L. (2012). Autophagy-mediated HMGB1 release antagonizes apoptosis of gastric cancer cells induced by vincristine via transcriptional regulation of Mcl-1. Autophagy 8 (1), 109–121. 10.4161/auto.8.1.18319 22108005

[B143] Zhang Y. Cheng Y. Ren X. Zhang L. Yap K. L. Wu H. (2012). NAC1 modulates sensitivity of ovarian cancer cells to cisplatin by altering the HMGB1-mediated autophagic response. Oncogene 31 (8), 1055–1064. 10.1038/onc.2011.290 21743489 PMC3275651

[B144] Zhang Q. Si S. Schoen S. Chen J. Jin X.-B. Wu G. (2013). Suppression of autophagy enhances preferential toxicity of paclitaxel to folliculin-deficient renal cancer cells. J. Exp. Clin. Cancer Res. 32, 99. 10.1186/1756-9966-32-99 24305604 PMC3879005

[B145] Zhang S. F. Wang X. L. Yang X. Q. Chen N. (2014). Autophagy-associated targeting pathways of natural products during cancer treatment. Asian Pac. J. Cancer Prev. 15 (24), 10557–10563. 10.7314/apjcp.2014.15.24.10557 25605139

[B146] Zhang X. Li W. Wang C. Leng X. Lian S. Feng J. (2014). Inhibition of autophagy enhances apoptosis induced by proteasome inhibitor bortezomib in human glioblastoma U87 and U251 cells. Mol. Cell Biochem. 385 (1-2), 265–275. 10.1007/s11010-013-1835-z 24104452 PMC3840293

[B147] Zhang D. Tang B. Xie X. Xiao Y. F. Yang S. M. Zhang J. W. (2015). The interplay between DNA repair and autophagy in cancer therapy. Cancer Biol. Ther. 16 (7), 1005–1013. 10.1080/15384047.2015.1046022 25985143 PMC4622693

[B148] Zhang R. Wang R. Chen Q. Chang H. (2015). Inhibition of autophagy using 3-methyladenine increases cisplatin-induced apoptosis by increasing endoplasmic reticulum stress in U251 human glioma cells. Mol. Med. Rep. 12 (2), 1727–1732. 10.3892/mmr.2015.3588 25846607 PMC4464427

[B149] Zhang H.-Q. Fang N. Liu X.-M. Xiong S.-P. Liao Y.-Q. Jin W.-J. (2015). Antitumor Activity of Chloroquine in Combination with Cisplatin in Human Gastric Cancer Xenografts. Asian Pac. J. Cancer Prev. 16 (9), 3907–3912. 10.7314/apjcp.2015.16.9.3907 25987058

[B150] Zhao D. Yuan H. Yi F. Meng C. Zhu Q. (2014). Autophagy prevents doxorubicininduced apoptosis in osteosarcoma. Mol. Med. Rep. 9 (5), 1975–1981. 10.3892/mmr.2014.2055 24639013

[B151] Zheng Z. Liu T. Zheng J. Hu J. (2017). Clarifying the molecular mechanism associated with carfilzomib resistance in human multiple myeloma using microarray gene expression profile and genetic interaction network. OncoTargets Ther. 10, 1327–1334. 10.2147/OTT.S130742 PMC5338971 28280367

[B152] Zhu Y. X. Jia H. R. Gao G. Pan G. Y. Jiang Y. W. Li P. (2020). Mitochondria-acting nanomicelles for destruction of cancer cells via excessive mitophagy/autophagy-driven lethal energy depletion and phototherapy. Biomaterials 232, 119668. 10.1016/j.biomaterials.2019.119668 31927179

[B153] Zou Y. Ling Y.-H. Sironi J. Schwartz E. L. Perez-Soler R. Piperdi B. (2013). The autophagy inhibitor chloroquine overcomes the innate resistance to erlotinib of non-small cell lung cancer cells with wild-type EGFR. J. Thorac. Oncol. 8 (6), 693–702. 10.1097/JTO.0b013e31828c7210 23575415 PMC3855301

